# Stem/progenitor cells in pituitary organ homeostasis and tumourigenesis

**DOI:** 10.1530/JOE-17-0258

**Published:** 2017-08-30

**Authors:** Scott Haston, Saba Manshaei, Juan Pedro Martinez-Barbera

**Affiliations:** Developmental Biology and Cancer Research ProgrammeBirth Defects Research Centre, UCL Great Ormond Street Institute of Child Health, London, UK

**Keywords:** pituitary, stem cell, pituitary adenoma, homeostasis, adamantinomatous craniopharyngioma

## Abstract

Evidence for the presence of pituitary gland stem cells has been provided over the last decade using a combination of approaches including *in vitro* clonogenicity assays, flow cytometric side population analysis, immunohistochemical analysis and genetic approaches. These cells have been demonstrated to be able to self-renew and undergo multipotent differentiation to give rise to all hormonal lineages of the anterior pituitary. Furthermore, evidence exists for their contribution to regeneration of the organ and plastic responses to changing physiological demand. Recently, stem-like cells have been isolated from pituitary neoplasms raising the possibility that a cytological hierarchy exists, in keeping with the cancer stem cell paradigm. In this manuscript, we review the evidence for the existence of pituitary stem cells, their role in maintaining organ homeostasis and the regulation of their differentiation. Furthermore, we explore the emerging concept of stem cells in pituitary tumours and their potential roles in these diseases.

## Introduction

**Figure 1:**
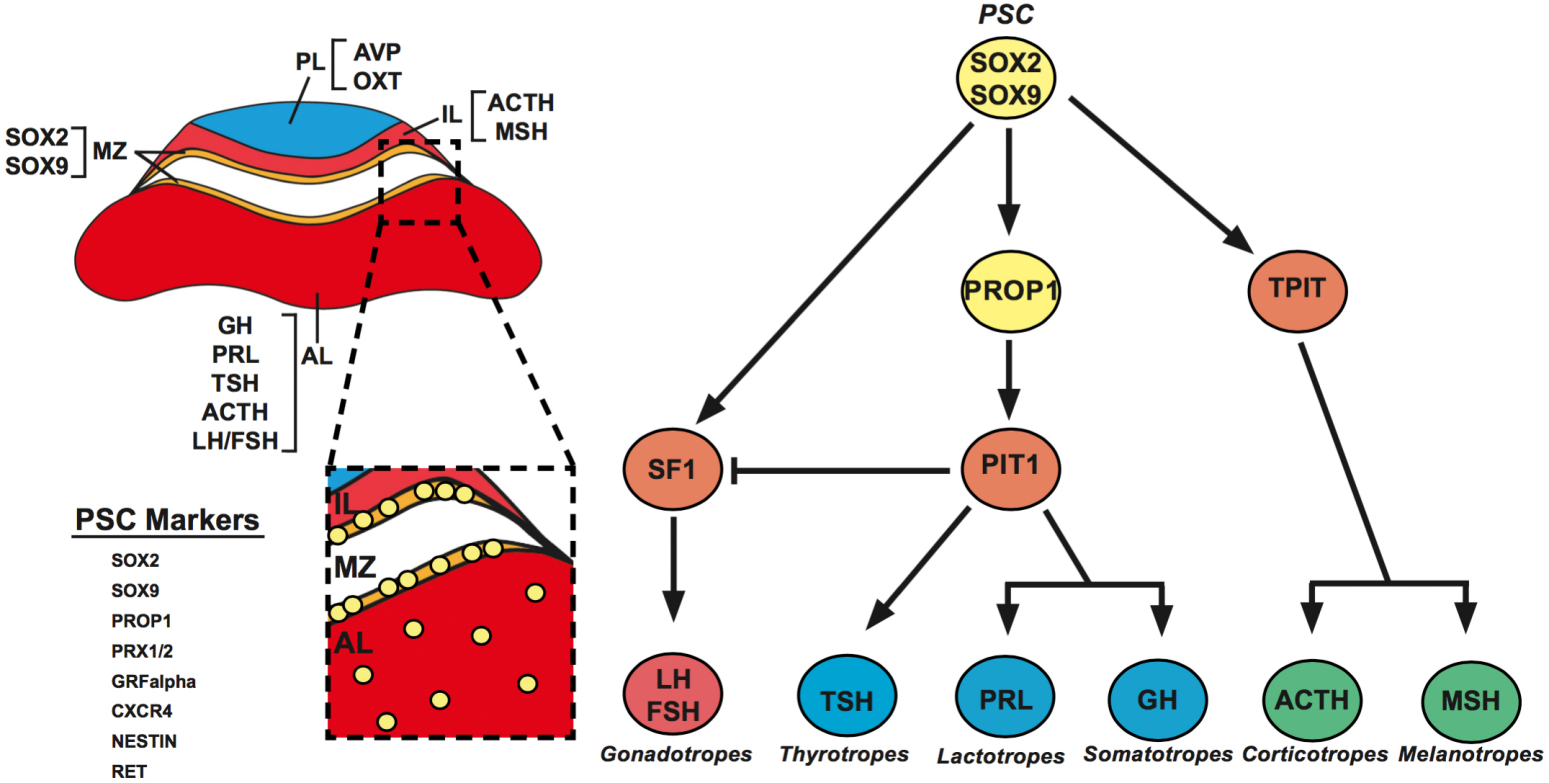
*Left*: Schematic representation of a coronal section through an adult mouse pituitary. Anatomical locations of the hormone-expressing cells are indicated in the posterior (PL), intermediate (IL) and anterior (AL) lobes. The PL contains the axons of the hypothalamic magnocellular neurosecretory neurons that release vasopressin (AVP) and oxytocin (OXT). The IL contains melanotrophs and corticotrohs. The AL holds the somatotrophs, lactotrophs, thyrotrophs, corticotrophs and gonadotrophs. PSCs are concentrated in the MZ, at the dorsal region of the AL lining the cleft, and scattered throughout the AL. These cells can be identified through the expression of several PSC markers. *Right*: A genetic pathway showing the cellular hierarchy of differentiation in the pituitary with essential transcription factors, which regulate lineage commitment.

The pituitary gland is known as the master regulator of the endocrine system, a title that is justified by the numerous critical physiological functions that it regulates including growth, metabolism, stress responses and reproduction. The organ is composed of anterior (AL), intermediate (IL) and posterior lobes (PL), with the former two being derived from Rathke’s pouch, an invagination of the oral ectoderm and the latter being a derivative of the overlying diencephalic neural ectoderm. The AL contains five distinct hormone-producing cell types: somatotrophs, thyrotrophs, lactotrophs, corticotrophs and gonadotrophs, which secrete growth hormone (GH), thyroid-stimulating hormone (TSH), prolactin (PRL), adrenocorticotropin (ACTH) and gonadotropins (FSH and LH), respectively (Kelberman *et al.* 2009). These secretory cells have been defined by the expression of specific-lineage transcription factors that are important for their induction and maintenance. These lineage commitment markers include Sf1, which directs differentiation of the gonadotroph cell lineage (Schimmer & White 2010), Tpit, which activates proopiomelanocortin (*Pomc1*) in corticotrophs (Lamolet *et al.* 2001), and Pit-1, which results in the production of lactotrophs, thyrotrophs and somatotrophs (Dollé *et al.* 1990). These hormonal lineages are generated during embryonic development and are all specified by birth (Japón *et al.* 1994) (Fig. 1). Interestingly, Pit-1-independent differentiation of a transient population of thyrotrophs has been observed in the rostral tip of Rathke’s pouch, which becomes the pars tuberalis of the pituitary. These cells arise around embryonic day 12, preceding the formation of the mature Pit-1-dependant thyrotrophs that persist into adult life and are lost by the time of birth (Lin *et al.* 1994).

The pituitary possesses a remarkable capacity for plasticity in regulating the number and proportion of secretory cell types in response to physiological changes, such as puberty and pregnancy (Melmed 2003, Perez-Castro *et al.* 2012). Recently, it has been demonstrated that the AL contains populations of stem/progenitor cells, which contribute to the production of hormone-producing cells during development and postnatal life (Garcia-Lavandeira *et al.* 2015). Stem-like cells have also been identified from pituitary adenomas and other pituitary neoplasias raising the possibility that they represent a tumour-initiating cell population. The elucidation of the mechanisms underlying pituitary stem cell (PSC) self-renewal, differentiation and programmed death may lead to a greater understanding of pituitary homeostasis, physiological plasticity and tumorigenesis. Ultimately, this may inform future translational research on hypopituitarism and neoplasia. This review aims to explore our current understanding of the identity of PSCs, particularly in the adult context, as well as their role in maintaining organ homeostasis and contribution to tumorigenesis.

## Pituitary stem cells during embryonic and postnatal life

### Identification of adult pituitary stem cells

Resident tissue-specific stem cells are found in most organs, where they are critical for normal homeostasis. Stem cells reside in specialised microenvironments known as the niche, which provides molecular cues to maintain stemness and direct their differentiation into transit-amplifying and/or terminally differentiated somatic cells. Stem cells are also characterised by their ability to self-renew, maintaining a long-term pool of undifferentiated progenitors for future rounds of differentiation (Hsu *et al.* 2014). Therefore, stem cells are able to provide the means for a tissue to maintain homeostatic balance and regeneration following injury (van Es *et al.* 2012, Patel *et al.* 2013).

Convincing evidence of the existence of adult PSCs has been gathered over the last decade. These PSCs are thought to reside in the IL, dorsal AL, a region known as the marginal zone (MZ) and dispersed throughout the AL parenchyme. Work by Lepore in 2005 demonstrated that the culture of dissociated pituitary tissue in stem cell-promoting media resulted in the generation of adherent colonies, which express S100β and have the capacity to differentiate into hormone-producing cells (Lepore *et al.* 2005). Similar experiments later showed that if dissociated pituitaries are grown in non-adherent conditions, ‘pituispheres’ can be generated, which efflux verapamil-sensitive Hoechst dye, allowing for their identification by flow cytometry (Chen *et al.* 2005). Further work uncovered that this side population expresses the markers SOX2, SOX9, CD4, CD133 and stem cell antigen-1 (SCA1) (Chen *et al.* 2009).

Others employing the use of *in vitro* clonogenicity assays have uncovered pituitary cells that express PROP1, PRX1/2, GFRα2, CXCR4 and NESTIN and possess clonogenic potential (Gleiberman *et al.* 2008, Garcia-Lavandeira *et al.* 2009, Nomura *et al.* 2009, Horiguchi *et al.* 2012, Rizzoti *et al.* 2013, Higuchi *et al.* 2014). NESTIN was also demonstrated to be co-expressed with SOX2 in cells of the MZ; however, its expression is known to be heterogeneous as NESTIN is also expressed in non-hormonal pituitary cells, making it unsuitable as a definitive marker of PSC (Krylyshkina *et al.* 2005, Vankelecom 2007). PROP1, a transcription factor that is indispensable for pituitary development, has also been found to be expressed in putative PSC populations and has been associated with stemness as its downregulation, along with SOX2, is required for hormonal cell differentiation (Fauquier *et al.* 2008, Chen *et al.* 2009, Garcia-Lavandeira *et al.* 2009, Yoshida *et al.* 2009, Gremeaux *et al.* 2012). Moreover, PROP1 has been shown to be required to maintain normal numbers of undifferentiated PSCs (Pérez Millán *et al.* 2016). Further work analysing PSC side populations has revealed that they express the paired homeodomain protein PRX1 and PRX2, which have been suggested to have functions in the proliferation and maintenance of embryonic pituitary progenitors in Rathke’s pouch (Vankelecom 2010, Susa *et al.* 2012). Expression of the chemokine receptor CXCR4 and its ligand, CXCL12, have also been identified in PSC side populations, along with other cells of the AL, with suggestions that this signalling axis may contribute to maintenance and migration of PSCs (Barbieri *et al.* 2007, Vankelecom 2010, Horiguchi *et al.* 2012). Work by Garcia-Lavandeira has shown that a population of cells located in the MZ coexpress GFRα2, RET and PROP1 (referred to as ‘GPS’ cells), and it is suggested that these cells contribute to the stem cell niche in the MZ and may function in the regulation of structural guidance and survival of PSCs. Furthermore, this population was observed in both mouse and human pituitaries and found to coexpress the stemness markers SOX2 and SOX9 (Garcia-Lavandeira *et al.* 2009).

SOX2+ cells, which are negative for expression of hormonal markers, have been found predominantly localised to the MZ with scattered cells throughout the AL. SOX2 has been shown to be expressed in the clonogenic cells in both adherent and non-adherent cultures and isolation of SOX2+ cells by flow cytometry greatly enriched for colony-forming cells. Interestingly, only 1.5–5% of plated SOX2+ cells were capable of colony formation, indicating that the population is most likely heterogeneous, with only a subpopulation of cells possessing self-renewing capacity (Fauquier *et al.* 2008, Andoniadou *et al.* 2012). In the AL, SOX2 is predominantly co-expressed with SOX9, with partial overlap of S100β expression (Andoniadou *et al*. 2013, Rizzoti *et al.* 2013). Genetic lineage tracing experiments following the fate of SOX2+ cells in the pituitary *in vivo* showed that over long-term tracing (9 months) SOX2+ cells differentiated into all hormone-producing lineages. In addition, a proportion of traced cells express SOX2 and SOX9, but not any commitment or differentiation markers, suggesting self-renewal of the initially labelled SOX2+ cells (Andoniadou *et al.* 2013).

There is also evidence that supports the existence of a PSC population in humans. Pituispheres have been generated from five human pituitaries following autopsy and were shown to express the PSC markers NESTIN, LHX3 and PITX2. Furthermore, these spheres were shown to be able to express all pituitary hormones upon induction of differentiation (Weiss *et al*. 2009). Further work demonstrated the existence of a ‘GPS’ cell population in the human MZ, which colocalised with the stemness markers OCT4, KLF4, SOX2 and SOX9, a situation analogous to that in mouse (Garcia-Lavandeira *et al.* 2012).

In the past 10 years, there have been great strides in characterising putative PSC populations in both mice and humans. The use of flow cytometry to isolate side populations has provided a plethora of makers of PSC. Clonogenic assays have demonstrated the capability of these cells to both self-renew and differentiate in a multipotent fashion *in vitro*. Genetic lineage tracing strategies have also provided insights into the existence of adult PSC *in vivo*, albeit based on the expression of only SOX2 and SOX9. Together these studies identify that populations of cells possessing stem cell characteristics appear to be predominantly, but not exclusively, localised to the MZ. The question remains, however, whether PSC are a single population with multipotent differentiation capacity or distinct populations with more restricted lineage commitments. Further research will be required to solve this question and determine the definitive markers of PSC identity.

### Regulation of pituitary stem cell differentiation: insights from embryonic development and organoids

The lack of definitive markers of PSCs has hampered the elucidation of the mechanisms underlying their differentiation *in vivo*, therefore, limiting our understanding to the analysis of cell populations *in vitro*. As discussed previously, putative PSC populations can be grown *in vitro*, as either adherent colonies or as free-floating spheres. These cells can then be differentiated into all hormone-producing cell types. This has been achieved by different means including culture in the presence of a mixture of growth factors, hormones or conditioned media from differentiated pituitary cell lines (Fauquier *et al.* 2008, Chen *et al.* 2009, Garcia-Lavandeira *et al.* 2009). Due to the heterogeneous nature of the additives used, it is not possible to determine the exact factors required or the order in which cells must be exposed to them to induce directed differentiation along specific hormonal lineages.

An approach that has been adopted to determine the mechanisms of differentiation is to study the embryonic development of the pituitary, with the assumption that similar mechanisms govern embryonic and adult PSC differentiation. Independent work from several groups have shown the importance of a multitude of signalling molecules and transcription factors in controlling Rathke’s pouch progenitor specification, proliferation and differentiation of all hormone-producing cells (Reviwed in Kelberman *et al.* 2009, Perez-Castro *et al.* 2012). Work from Sasai’s group in 2011 demonstrated the validity of such approach. They utilised mouse embryonic stem cell (ESC) aggregates to study pituitary organogenesis. They uncovered that they could induce Rathke’s pouch progenitors (PITX1A+/LHX3+) by combining ESC aggregates with either oral ectoderm (PITX1A+) or hypothalamic identity (RX+) in the presence of a Shh pathway agonist (Suga *et al.* 2011). Shh has also been shown to be required for Lhx3 expression in explant cultures of human pituitaries (Treier *et al.* 2001). Following this, they tested strategies to induce commitment to specific hormonal lineages. Somatotrophs were obtained after a long culture period in the presence of a β-catenin agonist, cortisol and insulin, albeit at a low efficiency, with less than 6% of progenitor cells becoming GH+. Corticotroph differentiation was induced more robustly (40% of progenitors became ACTH+) through the action of hypoxia and a Notch pathway inhibitor. These results have also been recapitulated using human ESC and induced pluripotent stem cells (iPSC) demonstrating conserved mechanisms of specification between species (Dincer *et al.* 2013).

### The role of adult pituitary stem cells during organ homeostasis

The regenerative capacity of the pituitary gland has been tested using transgenic mouse models, in which specific hormonal lineages in adult mice could be genetically ablated. This research has provided evidence for a role of stem/progenitor cells in repopulating the gland. Work by Vankelecom’s group, in which somatotrophs (GH+) were rendered susceptible to diphtheria toxin-mediated ablation, revealed that the elimination of this lineage led to expansion of the SOX2+ stem/progenitor compartment, with increased numbers of double SOX2+/GH+ cells (Fu *et al.* 2012). Further to this, they also demonstrated that the regenerative potential of the organ was age dependant, with older mice failing to recover their GH+ population following ablation (Willems *et al.* 2016). Interestingly, this reduced regenerative potential correlates with a reduction in PSC during ageing. The Vankelecom group also demonstrated similar regeneration and expansion of the stem/progenitor compartment when lactotrophs (PRL+) were specifically ablated (Fu & Vankelecom 2012). Both studies, however, failed to show quantitatively consistent re-population of the organ and did not utilise genetic tracing to demonstrate that it was PSC that differentiated during regeneration. In contrast to these results, experimental reduction of the number of corticotrophs (ACTH+), by Drouin’s group, found that the contribution of PSC was negligible and that regeneration was attributed to replication of existing corticotrophs (Langlais *et al.* 2013). Like the previous two studies, the work by Drouin’s group did not perform lineage tracing.

As previously mentioned lineage tracing of putative PSCs has been performed by both our own (SOX2+ cell lineage tracing) and Lovell-Badge’s group (SOX2+ or SOX9+ cell lineage tracing) (Andoniadou *et al.* 2013, Rizzoti *et al.* 2013). Together these results showed that SOX2+ PSC populations differentiate to give rise to all hormonal lineage and self-renew long term. Work by Lovell-Badge’s group also performed functional experiments, in which traced SOX2+ or SOX9+ cells could be induced to proliferate *in vivo* due to the effect of oestradiol and could differentiate to a corticotroph fate following acute adrenalectomy. Interestingly, the authors found that traced cells had a predisposition to becoming LH+ gonadotrophs and lactotrophs (PRL+), with a small number differentiating into somatotrophs (GH+) (Rizzoti *et al.* 2013). Together, this work demonstrates that adult PSCs in normal conditions both self-renew and differentiate to form all AL hormonal lineages; however, the number of differentiated cells produced is small indicating low levels of physiological turnover. Furthermore, indirect evidence suggests that PSCs can be recruited under injury conditions to facilitate regeneration of the organ. However, this contribution seems to be limited, therefore indicating that proliferation of differentiated cells is the most likely source of this plasticity in some contexts.

It is well established that the adult pituitary is an organ with a low cell turnover, estimated to be around 1.5% of cells per day in adulthood, which declines during the ageing process (Nolan *et al.* 1998, Levy 2002). Although in certain contexts such as during development, early postnatal life, puberty, pregnancy and lactation proliferation in the pituitary has been observed to increase (Melmed 2003). It has generally been assumed that these proliferating cells represent the terminally differentiated secretory cell populations. Indeed, all differentiated hormonal cells have been shown to possess the capacity to divide, with females showing a proliferation bias towards lactotrophs and males towards somatotrophs (Oishi *et al.* 1993). Evidence for the involvement of PSCs, as opposed to replication of existing hormonal cells, in pituitary plasticity during pregnancy and lactation is currently sparse. Work by Zhu *et al*. demonstrated the importance of the Notch signalling pathway in maintaining postnatal SOX2+ PSC self-renewal using a genetically modified mouse line, in which PSCs were depleted through loss of Notch signalling in PROP1-expressing cells. They showed that the numbers of proliferating SOX2+ cells did not increase during gestation and lactation, however, increased proliferation was observed in PIT1+ cells. Furthermore, they revealed similar proliferation dynamics in both PSC-deficient and wild-type contexts. From this they concluded that the SOX2+ PSC were dispensable for the plastic adaptation during pregnancy and lactation. However, by performing bilateral adrenalectomy, the same group showed that PSCs as well as PIT1+ and ACTH+ cells increased proliferation. Interestingly, in the PSC-depleted genetic context, similar increases in proliferation and numbers of ACTH+ cells were observed indicating that the PSC populations’ contribution to plasticity was limited. These authors also noted, however, that the SOX2+ PSCs are important for postnatal expansion of the gland, as the proliferative index of the organ was halved in their Notch ablated, PSC-deficient context (Zhu *et al.* 2015). A recent study, however, reported the expansion of PSC during gestation and at the onset of lactation, but without experimental lineage tracing of PSCs, it is difficult to fully assess their involvement in meeting changing hormonal demands (Vaca *et al.* 2016). Analysis of PSC markers in rat pituitaries during normal conditions revealed that the number of these cells is at its highest at birth and that a continuous decline occurs throughout adult life. Concomitant with this, proliferation in the gland also decreases with age (Garcia-Lavandeira *et al.* 2009, Yoshida *et al.* 2011). In mice, the numbers of SOX2+ cells also decline following birth (Gremeaux *et al.* 2012).

To gain a more complete understanding of pituitary homeostasis, it is necessary to assess the role of programmed cell death. Apoptosis in the adult pituitary has been shown to occur at a very low rate, with increases observed during pregnancy and the postpartum period (Kulig *et al.* 1999). Recently, a mechanism has been proposed in which the RET/Pit-1/Arf/p53 signalling axis contributes to the regulation of pituitary cell turnover by apoptosis. The RET receptor and its co-receptor GFRA2 have been shown to be expressed in PSC. Furthermore, RET is also expressed alongside another co-receptor GFRA1 in somatotrophs (Urbano *et al.* 2000, Japón *et al.* 2002). These signalling complexes are activated by GDNF, which is expressed in the AL of rat and human pituitaries. RET has been demonstrated to act as a ‘dependence receptor’ in somatotrophs, where signalling activity is crucial for the survival of these cells (Cañibano *et al.* 2007). Absence of signalling ligand results in RET complexing with caspase-3 and PLC-delta (PKCd). Following this, caspase-3 becomes activated, processing RET and PKCd, resulting in the phosphorylation of the transcription factors CREB and cEBPa, among others, which leads to excessive Pit-1 transcription (Cañibano *et al.* 2007, Diaz-Rodriguez *et al.* 2014). Pit-1 then induces transcription of p19-ARF, subsequent stabilisation and accumulation of p53 and apoptosis. Intriguingly the dependence of Pit-1 in this apoptotic signalling network creates an association with the differentiation of PSC. Further *in vivo* evidence for the RET/Pit-1/Arf/p53 pathway can be observed in RET-knockout mice, which results in expanded somatotroph numbers causing pituitary hyperplasia (Cañibano *et al.* 2007). Moreover, it has been observed in normal pituitaries that most of the non-apoptotic (TUNEL-negative) and p19-ARF-negative somatotrophs show phosphorylation of the RET receptor, which is indicative of GDNF signalling activity. There is also some evidence that this apoptotic pathway is at play in other hormonal lineages. Following the cessation of lactation, there is an increased number of RET/activated Caspase-3 double positive cells, although the study did not determine whether these cells were lactotrophs (Guillou *et al.* 2011). There is also evidence for redundancy in AL programmed cell death mechanisms as autocrine production of dopamine has been found to be pro-apoptotic in lactotrophs following lactation (Jaubert *et al.* 2007). Together, these studies provide evidence for the existence of mechanisms of programmed cell death in terminally differentiated secretory cells of the pituitary, which are necessary for regulating cell number in response to physiological demand.

## The role of stem cells during pituitary tumorigenesis

### Cancer stem cells and tumour heterogeneity

Research over the past few decades has established that, generally, a tumour mass is not a homogenous environment, but rather it consists of a range of cell subpopulations varying in genetic, epigenetic and phenotypic properties (Marusyk & Polyak 2010, Burrell *et al.* 2013). This phenomenon, termed intra-tumour heterogeneity, can be explained through the cancer stem cell (CSC) model, whereby tumorigenesis follows a similar mode of development as organogenesis, in a hierarchical manner (Visvader & Lindeman 2008, Shackleton *et al.* 2009, Kreso & Dick 2014). This model proposes that a small population of cancerous cells with stem-like properties, termed CSCs, differentiate and through clonal evolution and environmental differences, make up a heterogeneous tumour bulk (Visvader & Lindeman 2008, Shackleton *et al.* 2009).

CSCs, similar to normal stem cells, are thought to have the ability to be maintained by a specific niche, self-renew and differentiate into all cell types within the tumour (Visvader & Lindeman 2008, Shackleton *et al.* 2009, Nguyen *et al.* 2012). In this model, these cells initiate and maintain tumorigenesis, yet, may be more resistant to chemotherapy and radiotherapy than other tumour cells (Abdullah & Chow 2013, Agliano *et al.* 2017). This resistance is due to their often (but not always) quiescent or slow cycling states (Stange & Clevers 2013, Martinez-Barbera & Andoniadou 2016) or the expression of ABC transporters and high levels of aldehyde dehydrogenases (Abdullah & Chow 2013).

CSCs were first identified in leukaemia, where it was observed that only a small population of the cancerous cells were capable of colony formation *in vitro* and cancer propagation *in vivo* (Bruce & Van Der Gaag 1963, Park *et al.* 1971, Sabbath *et al.* 1985, Griffin *et al.* 1986). These cells have since been isolated from solid tumours, including breast, brain, colorectal and pancreatic cancers (Southam & Brunschwig 1961, Hamburger & Salmon 1977, Al-Hajj *et al.* 2003, Visvader & Lindeman 2008). The exact origin of CSCs remains elusive. Some studies suggest that CSCs arise from tissue-specific stem cells following an oncogenic transformation (Bonnet & Dick 1997, Barker *et al.* 2009, Zomer *et al.* 2013), while others have identified that the cells can arise from differentiated cells through reprogramming and dedifferentiation (Griffin *et al.* 1986, Holczbauer *et al.* 2013, Friedmann-Morvinski & Verma 2014). Irrespective of their origin, CSCs are of high clinical significance, as it is believed that only through eradication of this population will the cancer be treated and relapse prevented.

### Cancer stem cells in pituitary adenomas

Although there has been extensive research in the field of CSCs over the past few decades, evidence for the presence of these cells in pituitary adenomas (PA) has only recently emerged (Carreno *et al.* 2016). With a prevalence of 78–94 cases/100,000 people (Karavitaki 2012), these tumours of the sellar region are one of the most common intracranial neoplasms (Larkin & Ansorge 2000). PAs are generally histopathologically benign; yet, they can be locally invasive and clinically relevant, causing endocrine disorders due to the overproduction of hormones. A proportion of them can grow enormously (giant PAs) and develop resistance to treatment (Larkin & Ansorge 2000, Chanson & Salenave 2004, Chatzellis *et al.* 2015, Carreno *et al.* 2016).

PAs represent a varied group of tumours, can be familial or sporadic, and are classified according to their endocrinological activity (hormone-secreting or non-functional), clinical manifestation, driver mutations and histological features (Larkin & Ansorge 2000, Di Ieva *et al.* 2014, Syro *et al.* 2015, Carreno *et al.* 2016). These tumours are generally recognised as monoclonal tumours, through evidence from X-chromosome inactivation and allelotype analyses (Alexander *et al.* 1990, Herman *et al.* 1990, Schulte *et al.* 1991, Ma *et al.* 2002). However, some studies have challenged this idea, having identified different clonality pre- and post-recurrence or between primary tumours and their metastases in rare malignant cases (Clayton *et al.* 2000, Clayton & Farrell 2001, 2004, Buch *et al.* 2002).

A number of studies have isolated cells within human and murine PAs (Xu *et al.* 2009, Hosoyama *et al.* 2010, Chen *et al.* 2014, Donangelo *et al.* 2014, Mertens *et al.* 2015), fulfilling some or all of the CSC criteria as follows: (1) clonogenic ability *in vitro*, (2) stem cell marker expression (e.g. CD133, CD166, SOX2, SCA1), (3) multipotency, (4) resistance to chemotherapeutic agents, (5) ability to initiate *de novo* tumours when transplanted into immunocompromised hosts (Welte *et al.* 2010, Martinez-Barbera & Andoniadou 2016). Other properties such as their ability to efflux Hoechst 33342 DNA-binding dye (through ABC transporters (Abdullah & Chow 2013) have also been used as an identifying marker of these cells (Chen *et al.* 2014), similar to PSC as previously described.

The first identification of CSCs from PA were reported by Xu and coworkers, whereby they isolated a population of cells from hormone-producing and non-producing benign adenomas, fulfilling most of the criteria above (Carreno *et al.* 2016, Xu *et al*. 2009). These cells were able to form floating sphere colonies similar to those formed by adult PSC (Chen *et al.* 2005), when grown in serum-free stem cell-promoting media. They also expressed pituitary specific markers such as PIT1 and markers of ‘stemness’ such as OCT4, NOTCH4, CD133 and NESTIN. Furthermore, they displayed chemotherapeutic resistance, could differentiate into hormone-producing cells in culture and initiated pituitary tumours when serially transplanted into NOD/SCID mice. Chen and coworkers were able to show similar results through isolation of CD133 and NESTIN-expressing cells from PA, which were able to initiate synaptophysin-positive tumours when xenotransplanted subcutaneously (Chen *et al.* 2014).

Other studies have also found populations within PAs with CSC properties. Work by Mertens and coworkers reported the identification of a side population within human adenomas that, when purified of endothelial and immune cells, expressed stemness markers such as SOX2, NESTIN and CXCR4 and epithelial–mesenchymal transition-linked factors (Mertens *et al.* 2015). This cell population had clonogenic, sphere-forming potential in culture, could be serial passaged and differentiated into hormone-producing cells. In contrast to previous studies, these human adenoma cells failed to expand when xenografted into SCID mice; however, they could identify a similar side population in the AtT20 pituitary tumour cell line, which expressed SOX2 and CXCR4, and can form *de novo* tumours in xenograft transplantations.

Supporting these results, Würth and coworkers recently published a study reporting the isolation of CSC-like cell populations from 38 human PA tumours (Wurth *et al.* 2016). These cells were CD133+, had clonogenic potential and could form spheres *in vitro*. They also expressed well-known stem cell markers such as OCT4, SOX2 and NESTIN and had the potential to differentiate into hormone-producing cells. Similar to the previously mentioned study, no pituitary tumour formation was observed in immunodeficient mouse xenograft experiments. However, it was shown that the human PA stem cells, when grafted into zebrafish embryos were pro-angiogenic and invasive, demonstrating their CSC-like properties in this alternative *in vivo* model (Wurth *et al.* 2016).

More recently, Manoranjan and coworkers compared and characterised PAs with high and low expression of CD15, a neural stem cell marker for CSCs in other brain tumours (Mao *et al.* 2009, Read *et al.* 2009). It was found that PAs that were enriched for CD15, also expressed SOX2 and PAX7 to a significantly higher degree than the CD15^Low^ tumours. When isolated, the CD15+ cell population was able to form spheres in culture and initiate pituitary tumours in mouse xenotransplant experiments (Manoranjan *et al.* 2016). Interestingly, it was also found that recurrent PAs contain a larger population of the CD15+ cells than other non-recurrent PAs, in agreement with other studies correlating numbers of CSCs within a tumour with its aggressiveness (Singh *et al.* 2003, Pallini *et al.* 2011).

The presence of CSCs does not preclude that other stem cells may exist within the tumours. For example, Orciani and coworkers identified and isolated CD133-expressing mesenchymal stem cells from growth hormone-expressing and non-secreting PA (Orciani *et al.* 2014). This subpopulation had osteogenic, chondrogenic and adipogenic differentiation potential and expressed ‘stemness’ markers such as OCT4 and NANOG. These cells, however, failed to differentiate into hormone-producing cells, and it was hypothesised that two different populations of stem cells might exist within pituitary tumours (Orciani *et al.* 2014). A study from Megnis and coworkers isolated a population of cells from hormone-secreting and non-secreting PAs that did not express markers of pituitary progenitor cells or CD133. The authors concluded that these cells were multipotent mesenchymal stromal cells, but not stem cells, due to their limited self-renewal potential (Megnis *et al.* 2016). Further studies are needed to determine whether these cell populations with CSC-associated characteristics may have a role in maintaining and supporting adenoma formation alongside pituitary cancer stem cells (Orciani *et al.* 2014, Megnis *et al.* 2016).

Finally, a recent study on the activation of the MAPK/ERK pathway during pituitary development has provided evidence for a population of proliferative SOX2-expressing cells within human papillary craniopharyngioma (PCP) tumours (Haston *et al*. 2017). PCPs are rare pituitary tumours, mostly occurring in adults, and generally harbouring BRAF p.V600E mutations (Brastianos *et al.* 2014). Although benign, they can be clinically aggressive and associated with high morbidity (Brastianos *et al.* 2014, Haston *et al*. 2017). It was shown that despite the expression of BRAF V600E protein throughout the tumour, only a small population of cells activated MAPK/ERK pathway. Furthermore, these cells did not express pituitary differentiation markers. Further characterisation revealed that these cells express SOX2 and represent the major proliferative cell population within human PCP. The study suggests that activated MAPK/ERK bestows proliferative capacity upon a small subpopulation of SOX2+ cells while impairing their differentiation. Further investigation into the nature of these cells is needed to verify the existence of a CSC population within human PCPs (Haston *et al*. 2017).

Overall, there has been rising evidence for the existence of a CSC population within PAs and other pituitary tumour types. Some studies have been able to characterise the potential CSCs in transplantation experiments (Xu *et al.* 2009, Chen *et al.* 2014, Manoranjan *et al.* 2016, Wurth *et al.* 2016), while others have only presented gene expression profiles and assessed their clonogenic and differentiation capacity (Mertens *et al.* 2015, Haston *et al*. 2017). There is also evidence for other types of CSCs within PA of a non-pituitary cell lineage origin (Orciani *et al.* 2014, Megnis *et al.* 2016). Once the presence of CSCs within pituitary tumours has been fully verified and characterised, effort should be made to target these cells to optimise treatment of pituitary neoplasms.

### Stem cells may non-cell-autonomously promote and maintain tumourigenesis in adamantinomatous craniopharyngioma

Adamantinomatous craniopharyngioma (ACP) is another type of benign yet clinically aggressive pituitary tumour of Rathke’s pouch origin (Andoniadou *et al.* 2012). These tumours are mostly paediatric and make up 5–11% of all of the brain tumours in children (Hölsken *et al.* 2016). Experiments in ACP mouse models have provided evidence that PSCs can initiate and promote tumourigenesis non-cell-autonomously, rather than cell-autonomously as proposed in the CSC model.

It has been found that the majority of ACP tumours harbour mutations in the *CTNNB1* gene, encoding β-catenin (Buslei *et al.* 2005). These mutations are thought to impair the proteosomal degradation of this protein, resulting in its nucleo-cytoplasmic accumulation and subsequent upregulation of the Wnt pathway (Buslei *et al.* 2005, 2007, Andoniadou *et al.* 2012, Martinez-Barbera 2015). These data were used to make the first genetically engineered mouse model (GEMM) of ACP by targeting Rathke’s pouch (RP) progenitors with a degradation-resistant form of β-catenin in *Hesx1^Cre/+^ ; Ctnnb1^lox(ex3)/+^* mice (Gaston-Massuet *et al.* 2011). The successful induction of tumours with similar properties to human ACP established that targeting RP progenitors with oncogenic β-catenin is sufficient to form tumours; hence, *CTNNB1* mutations are drivers of ACP tumourigenesis in mouse and humans (Gaston-Massuet *et al.* 2011, Martinez-Barbera 2015, Martinez-Barbera & Andoniadou 2016).

The ACP mouse model presented with clusters of cells accumulating nucleo-cytoplasmic β-catenin, an immunohistochemical hallmark of human ACP (Hofmann *et al.* 2006, Gaston-Massuet *et al.* 2011). Cluster cells expressed ‘stemness’ markers, such as SOX2, CyclinD2, p27^KIP1^ and NESTIN, did not proliferate and did not express any commitment or differentiation markers. The tumorigenic pituitaries in the ACP mouse model contained up to three times more cells with clonogenic potential when cultured in stem cell-promoting medium as compared to control pituitaries, signifying an expansion of the stem cell compartment (Gaston-Massuet *et al.* 2011). Whether CSCs are present in these mutant pituitaries remains as yet to be determined (Gaston-Massuet *et al.* 2011, Carreno *et al.* 2016, Martinez-Barbera & Andoniadou 2016). Interestingly, when mutant β-catenin was expressed in committed progenitors or differentiated hormone-producing cells, tumours did not form, suggesting that progenitors/stem cells may play a critical role in tumour induction. This led to the development of a second mouse model specifically targeting SOX2+ cells with oncogenic β-catenin in *Sox2^CreERT2/+^; Ctnnb1^Lox(ex3)/+^* mice (Andoniadou *et al.* 2013). This model also developed synaptophysin-negative tumours and presented with nucleocytoplasmic β-catenin-accumulating cell clusters.

**Figure 2:**
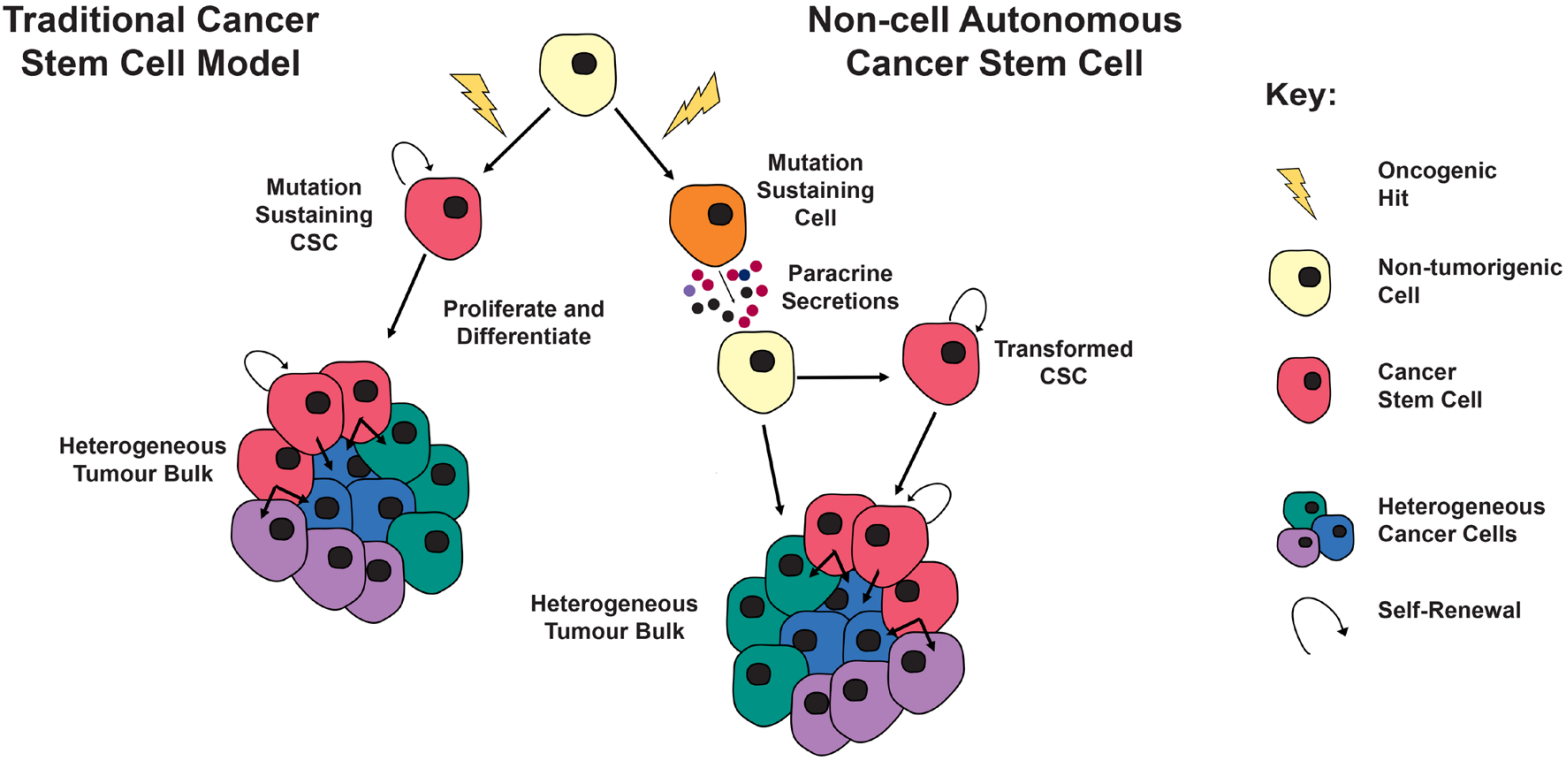
*Left*: The CSC model posits that the mutation-sustaining cells, a stem cell, committed progenitor or differentiated cell, generate progeny that constitute the bulk of the tumour cell-autonomously (red cells). These CSCs are able to persist in the tumour to continuously give rise to new tumour cells (green, blue and purple cells), similar to the role of somatic stem cells in normal tissue homeostasis. *Right*: The non-cell autonomous model of tumorigenesis differs in that the mutation-sustaining cell (orange cell) is not the cell of origin for the tumour. Instead this mutated cell, through paracrine interactions, causes transformation of a neighbouring cell (yellow cell) that can cell-autonomously generate the tumour, either though simple clonal expansion or perhaps through the generation of CSCs (red cells), which give rise to a tumour, as per the canonical cancer stem cell model.

Interestingly, when genetic lineage traced with a YFP reporter, it was shown that cluster cells originated from the SOX2+ cells targeted with mutant β-catenin (Andoniadou *et al.* 2013). However, the tumour bulk was found to be YFP negative, suggesting that tumour cells had a different cell of origin to the mutation-sustaining SOX2+ cells. This is in contrast to the CSC model, where the tumour is clonally derived from the initial mutation-sustaining cell (Carreno *et al.* 2016) (Fig. 2).

Molecular studies revealed that cluster cells from the ACP mouse models showed distinctive expression of several chemokines, cytokines and growth factors (Andoniadou *et al.* 2012, 2013), suggesting a potential paracrine model of tumour induction and maintenance. In this model, the oncogene-sustaining stem cells induce the formation of CSCs, which act as the tumour cell of origin giving rise to the bulk of tumour cells (Andoniadou *et al.* 2013, Martinez-Barbera & Andoniadou 2016). It was proposed that these paracrine signals could be acting on the cells directly or indirectly by promoting tumorigenesis through changes in the microenvironment (Andoniadou *et al.* 2013, Martinez-Barbera & Andoniadou 2016).

Although still an emerging field, non-cell autonomous induction and promotion of tumour formation has previously been described in a number of cases (Nicolas *et al.* 2003, Park *et al.* 2011, Laberge *et al.* 2012, Deschene *et al.* 2014, Plaks *et al.* 2015, Di Mitri & Alimonti 2016, Mescher *et al.* 2017). There is an opportunity for more research to characterise the exact role of these β-catenin-accumulating cell clusters and their paracrine secretions in mouse and human ACP.

## Concluding remarks

The pituitary gland, also referred to as the master gland, is the organ responsible for the regulation of critical endocrine functions. There is now evidence for a population of stem cells within this organ, which are mostly active during development and early postnatal life. These PSC may also contribute to the organ’s regenerative capacity as well as its plastic nature, participating in the homeostatic balance of hormone-producing cells through different physiological events.

Over the past decade, there has been extensive research characterising this PSC population in mice and humans. Lineage tracing has shown evidence of adult PSCs capable of differentiation into all hormone-producing cell types *in vivo* and flow cytometric analyses have identified markers for this cell population. Clonogenic assays have verified the isolated cell populations’ ability to self-renew and differentiate *in vitro*. The regenerative capacity of PSC has also been explored through selective ablation of specific pituitary hormonal lineages. However, due to the lack of lineage tracing, it is difficult to definitively assess whether the PSCs are responsible for replenishing the ablated populations. Unresolved questions remain, such as whether there is a single population with multipotent differentiation potential within the pituitary or distinct populations with more restricted commitments. Furthermore, recent advances in characterising the molecular markers of PSC will facilitate the elucidation of the molecular mechanisms underlying their differentiation.

In addition to their role in normal physiology, there is now evidence for the involvement of PSCs during tumorigenesis. Many studies have shown the presence of populations of undifferentiated cells with clonogenic ability within pituitary tumours suggesting that the CSC model may be relevant to pituitary neoplasms. Finally, research on adamantinomatous craniopharyngioma has provided evidence for a non-cell autonomous mechanism of tumour formation, in contrast to the conventional CSC model. A greater understanding of PSC regulation in both physiological and pathological contexts will not only aid in improving our basic understanding, but will ultimately help design novel treatments against hypopituitarism and pituitary tumours.

## Declaration of interest

The authors declare that there is no conflict of interest that could be perceived as prejudicing the impartiality of this review.

## Funding

This work was supported by the Medical Research Council (MRC) (Grant MR/M000125/1, Children with Cancer UK (Grant 164402) and by the National Institute for Health Research Biomedical Research Centre at Great Ormond Street Hospital for Children National Health Service Foundation Trust and University College London. S H holds a Wellcome Trust PhD Fellowship. J P M-B is a Great Ormond Street Children Hospital Charity Principal Investigator.

## Author contribution statement

All authors contributed equally to the writing of the manuscript. S H and S M generated the figures.
